# Comparative Study of Intranasal Dexmedetomidine Versus Intranasal Ketamine as Premedicant in Children

**DOI:** 10.7759/cureus.26572

**Published:** 2022-07-05

**Authors:** Nidhi Arun, Annu Choudhary, Mukesh Kumar

**Affiliations:** 1 Anaesthesiology, Indira Gandhi Institute of Medical Sciences, Patna, IND; 2 Surgery, Indira Gandhi Institute of Medical Sciences, Patna, IND

**Keywords:** post-anaesthesia recovery, children, ketamine, dexmedetomidine, intranasal premedication

## Abstract

Background: Pre-operative anxiety in children not only makes induction difficult but it is also associated with an increase in the requirement of analgesics, the incidence of post-operative nausea and vomiting (PONV), emergence delirium (ED), and postoperative maladaptive behavioral changes. It can be reduced effectively by pharmacological interventions. In a quest to find the ideal premedicant and non-invasive way of its administration, we decided to compare intranasal (IN) dexmedetomidine with IN ketamine as a premedicant in pediatric patients.

Aims and objectives: To compare sedation score, mask acceptance score (MAS) during induction, the incidence of ED, and other adverse events in both groups.

Material and methods: Some 60 children, between 1 and 8 years of age of either sex undergoing surgical procedures were included in this study and randomly divided into two groups (Group D and Group K). Thirty minutes prior to induction of anesthesia, patients of Group D received dexmedetomidine 1 mcg kg^-1^ in 1 mL of 0.9% saline intranasally and patients of Group K received ketamine 5 mg kg^-1^ in 1 mL of 0.9% saline intranasally through calibrated dropper (0.5 mL in each nostril) in a recumbent position. Incidences of sneezing or coughing after IN administration of study drugs were recorded. The subsequent sedation scores were assessed using MOASS at 15 min, then at 30 min following premedication at the time of parental separation. After shifting patients to operation theater inhalation induction was done. MAS at induction and any adverse effects were recorded.

Results: Children in Group K were found to be significantly more sedated at 30 min after administration of premedication and mask acceptance was also better (p value < 0.0001 with a confidence interval, CI=95%). But the incidence of ED and PONV was high.

Conclusion: Intranasal dexmedetomidine (1 mcg kg^-1^) is clinically less effective as a premedicant in terms of sedation and mask acceptance in older children as compared to ketamine (5 mg kg^-1^), but associated with fewer incidence of ED and PONV. We recommend the usage of IN dexmedetomidine in a higher dose (1.5-2 mcg kg^-1^), through nebulization/atomizer for the desired level of sedation and mask acceptance.

## Introduction

The alien environment of a hospital, being surrounded by strangers in hospital attire, fear of separation from parents, fear of surgery, and pain can make any child extremely anxious in the pre-operative period. A fearful and agitated child is stressful for both anesthesiologists and parents. Pre-operative anxiety and fear not only make induction difficult but it is also associated with an increase in the requirement of analgesics, the incidence of post-operative nausea and vomiting (PONV), emergence delirium (ED), and postoperative maladaptive behavioral changes in children like sleep disturbances, aggressive behavior, eating disorder, nocturnal enuresis, etc. [1]

Preoperative counseling, behavioral interventions, parental presence while induction and good premedication are methods to alleviate peri-operative anxiety, smooth parental separation, and induction of anesthesia [2].

Ideal premedication should have a fast onset of action, short duration, and convenient route of administration, readily accepted by children, minimal side effects, analgesic properties, and ability to attenuate sympathetic responses [3].

Many sedative analgesic agents and routes of delivery for the facilitation of painful procedures have been studied, with varying degrees of patient acceptance, efficacy, and safety [4]. To date, there remains no widely accepted premedication regimen [5].

Ketamine has been used as an effective premedicant for years. However, it is far from an ideal premedicant due to its undesirable effects like paradoxical reactions, cognitive impairment, postoperative behavioral changes, and delayed recovery. The introduction of dexmedetomidine, which is a highly selective alpha 2-agonist with a shorter half-life, with properties to provide anxiolysis, sedation, and analgesia without respiratory depression has given us hope to end the search for an ideal premedicant in children [6]. Its bioavailability is 81.8% when administered via the nasal mucosa [7]. The intranasal (IN) route is an effective, non-invasive, well-tolerated, and rapid way to administer premedication in children.

In a quest to find the ideal premedicant and non-invasive way of its administration, we decided to compare IN dexmedetomidine with IN ketamine as a premedicant in pediatric patients. Our aims were to assess, document, and compare a) sedation score, b) level of parental separation anxiety, c) mask acceptance score (MAS) during induction, d) hemodynamic parameter during induction, e) incidence of ED, and f) any peri-operative adverse events in both groups.

## Materials and methods

Prior to the commencement of this randomized, comparative and double-blinded study, ethical approval was obtained from the institutional ethical committee. The trial was registered with Clinical Trial Registry, India [ctri.nic.in] vide registration number CTRI/2017/10/010269. Based on a previous study by Diwan et al., keeping alpha error <0.05 and beta error <0.2, power of study <0.001, 60 children, between 1 and 8 years of age of either sex undergoing surgical procedures, of American Society of Anesthesiology physical status (ASA-PS) grade I, were included in this study [8].

Careful pre-anesthetic examination and written informed consent were obtained from parents of all children, before enrolling them in the study. We excluded those children who were obese (body mass index, BMI > 30), or have either anticipated difficult airway, or associated liver or renal dysfunction or known allergy to study drugs, or who are drowsy on the day of surgery with Modified Observer Assessment of Alertness/Sedation Scale (MOASS) < 5, or with nasal mass, or history of nasal bleeding.

Standard fasting protocol was followed for all the children. Baseline sedation scores were recorded, using MOASS. Patients were divided into two groups (Group D and Group K), based on computer-generated table of random numbers.

Some 30 min prior to induction of anesthesia, patients of Group D received dexmedetomidine 1 mcg kg-1 in 1 mL of 0.9% saline intranasally and patients of Group K received ketamine 5 mg kg-1 in 1 mL of 0.9% saline intranasally through calibrated dropper (0.5 mL in each nostril) in a recumbent position. The study drug was prepared by one anesthesiologist as per the group allocation and administered by the other anesthesiologist who was blinded to the group allocation. Incidences of sneezing or coughing after IN administration of study drugs were recorded to assess the patient acceptance of medication by a blinded observer. All children were nursed in either supine or recumbent position in the caregiver’s lap and monitored for heart rate and oxygen saturation, in pre-operative holding area. The subsequent sedation scores were assessed using MOASS at 15 min, then at 30 min following premedication at the time of parental separation (Table 1).

**Table 1:** Modified observer assessment of alertness/sedation scale.

Score	Response
5	Responds readily to name spoken in normal tone
4	Lethargic response to name spoken in normal tone
3	Response only after name is called loudly and/or repeatedly
2	Response only after mild prodding or shaking
1	Response only after painful trapezius squeeze
0	No response after painful trapezius squeeze

After shifting patients to operation theater (OT), basic monitors like pulse oximeter and electrocardiogram (ECG) and non-invasive blood pressure (NIBP) monitors were attached. Baseline heart rate (HR) and oxygen saturation (SpO2) were recorded. Anesthesia was induced with oxygen (O2) and air (50:50%) with a high concentration of sevoflurane [3-4 minimum alveolar concentration (MAC) percentages] on spontaneous respiration (tidal volume breathing) using Jackson-Rees modification of Ayre’s T-piece (Mapleson F) circuit via face mask. MAS was recorded at this point (Table 2).

**Table 2:** Mask acceptance score.

Score	Response
1 (Good)	Patient allows mask over his face without any resistance
2 (Average)	Patient allows mask over his face with some resistance which can be overcome by the person holding the mask
3 (Poor)	Patient allows mask over his face with significant resistance, that cannot be overcome by the person holding the mask alone and requires additional help

Intravenous (IV) access was secured. Injection propofol 2 mg kg^-1^ and fentanyl 2 mcg kg^-1^ along with atracurium 0.6 mg kg^-1^ as a muscle relaxant, was used. An endotracheal tube (ETT) or a laryngeal mask airway (LMA) of appropriate size was used to secure the airway. Maximum and minimum HR at induction was recorded. Induction time was defined as the time from the application of the mask till securing the airway. At the end of the surgery, after the reversal of general anesthesia, emergence agitation (EA) was assessed using the Watcha scale (Table 3).

**Table 3:** Watcha scale for emergence delirium.

Score	Response
0	Asleep
1	Calm
2	Crying but can be consoled
3	Crying but cannot be consoled
4	Agitated and thrashing around

A score of more than 2 indicates the presence of EA. Patients with agitation scores of more than 2 received IV midazolam (0.01-0.02 mg kg^-1^). In the post-anesthesia care unit (PACU), the patients were monitored according to PACU protocol. The incidence of peri-operative adverse events such as hypotension, bradycardia, tachycardia, and PONV was also recorded.

Descriptive statistics like mean and standard deviation have been used for continuous variables. Comparisons between the study groups were conducted using Pearson’s Chi-square test. The changes in BP and HR from baseline among the groups were tested by student’s t-test. The confidence interval (CI) was kept at 95% with a degree of freedom of 58.

## Results

**Figure 1:**
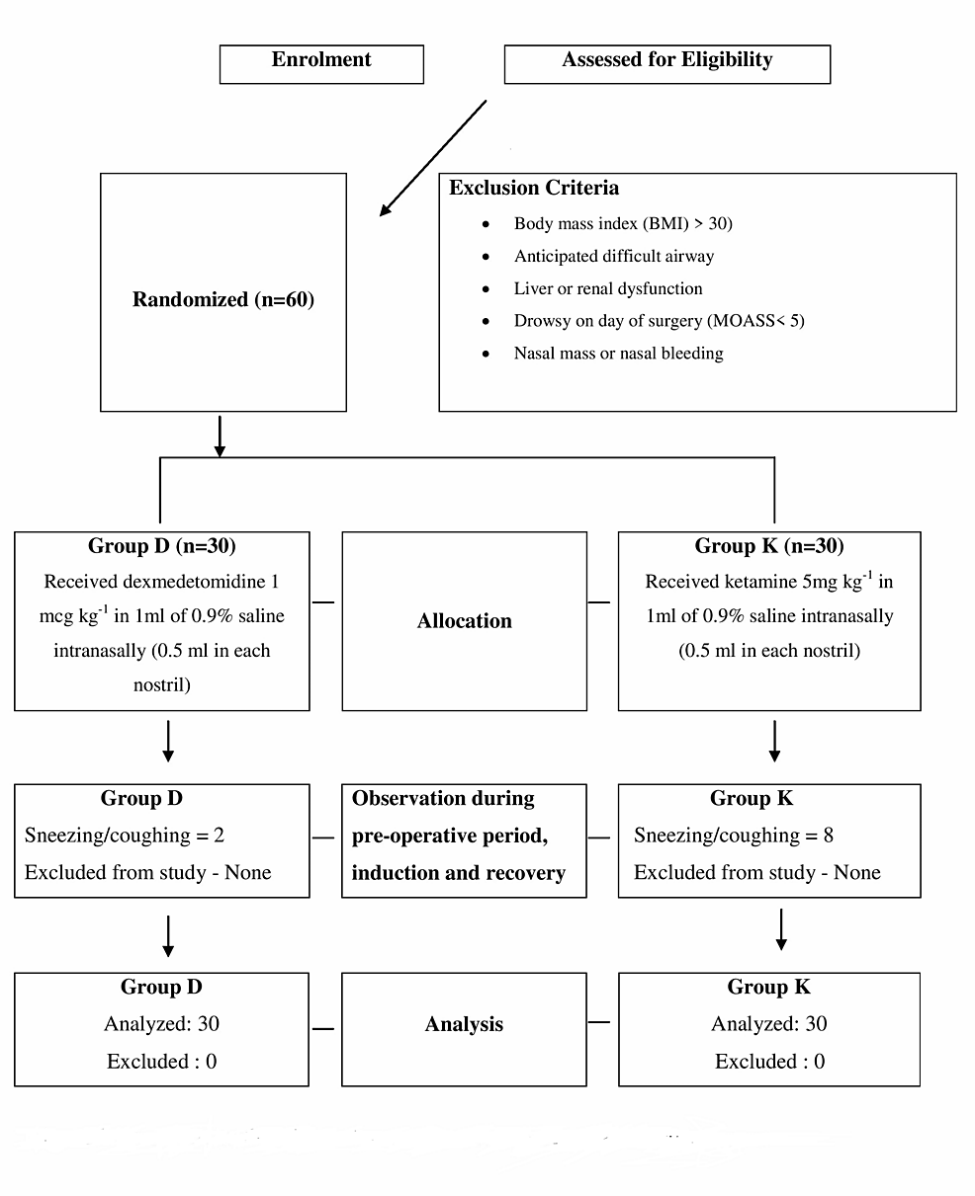
Simplified methodology of conduction of study shown as consort diagram.

A total of 60 patients were enrolled and randomly allocated into two groups in this study; various pre-decided outcome parameters were recorded and analyzed, as shown in the consort diagram (Figure 1). 

Patients of both groups were comparable with respect to age, sex, and weight (Table 4).

**Table 4:** Demographic parameter.

S. No.	Parameter	Group D (Mean ± SD)	Group K (Mean ± SD)	p value
1.	Age (years)	5.14 ± 1.60	4.75 ± 1.48	0.34
2.	Gender (female: male)	23:07	25:05	0.757
3.	Weight (kg)	13.87 ± 4.71	12.07 ± 4.06	0.118

All children remained hemodynamically stable and no airway compromise or fall in SpO2 below 94% requiring oxygen supplementation was observed in pre-operative holding area (Tables 5-6).

**Table 5:** Mean heart rate at different time intervals in pre-operative holding area.

S. No.	Mean heart rate (min)	Group D (Mean ± SD)	Group K (Mean ± SD)	p value
1.	0	97.0 + 10.95	98.5 +10.37	0.580
2.	5	115.7 + 12.67	114.7 + 13.96	0.773
3.	10	105.8 + 11.45	107.3 +12.86	0.635
4.	15	99.8 + 10.92	100.6 + 10.87	0.759
5.	20	95.3 + 11.60	98.2+ 12.07	0.341
6.	25	89.4 + 11.84	95.5 + 13.54	0.070
7.	30	88.1 + 10.01	89.8 + 11.86	0.559

**Table 6:** Mean oxygen saturation at different time intervals in pre-operative holding area.

S. No.	Mean SpO2 (min)	Group D (Mean ± SD)	Group K (Mean ± SD)	p value
1.	0	99.2 + 1.12	99.1 + 1.01	0.810
2.	5	98.8 + 1.13	99.0 + 1.01	0.475
3.	10	99.4 + 0.93	98.8 + 1.13	0.052
4.	15	99.6 + 0.81	99.4 + 0.89	0.549
5.	20	99.2 + 0.99	99.5 + 0.86	0.171
6.	25	99.6 + 0.81	99.5 + 0.81	0.875
7.	30	99.8 + 0.61	99.7 + 0.69	0.694

The baseline mean sedation score was 5 on MOASS in both groups. After IN administration of the study drug in the pre-operative area, the sedation score was assessed at 15 and 30 min intervals. The mean sedation score at 15 min (SS15) in Group D was found to be 4.1 ± 0.59, whereas SS15 in Group K was recorded at 4.1 ± 0.37. Both values were comparable (p value=0.6423 with CI=95% and t=0.466). Children in Group K were found to be significantly more sedated at 30 min after administration of IN premedication (p value < 0.0001 with CI=95% and t=11.70). The mean sedation score at 30 min (SS30) in Group K was recorded as 2.5 ± 0.5, whereas in Group D was 3.83 ± 0.372. After 30 min all children were shifted to OT. Mask acceptance was also found to be significantly better in Group K (p<0.0001 with CI=95% and t=11.83). Mean MAS in Group K was 1.3 ± 0.46 and in Group D was 2.7 ± 0.46. At the time of induction minimum and maximum HR were recorded in both groups. Children in Group D had comparatively lower mean minimum HR 78.9 ± 7.34) than in Group K (112.57 ± 14.63). Similarly, mean maximum HR was also lower in Group D (112.9 ± 14.69) than in Group K (133.93 ± 13.298). This difference was found to be statistically significant (p<0.0001) (Table 7).

**Table 7:** Observations.

S. No.	Parameter	Group D (Mean ± SD)	Group K (Mean ± SD)	t value	p value
1.	Sedation score at 15 min	4.10 ± 0.59	4.16 ± 0.37	0.47	0.6423
2.	Sedation score at 30 min	2.50 ± 0.50	3.8 ± 2.00	11.71	<0.0001
3.	Mask acceptance score	1.30 ± 0.46	2.7 ± 0.46	11.83	< 0.0001
4.	Minimum heart rate at induction	78.9 ± 7.34	112.57 ± 14.64	11.26	< 0.0001
5.	Maximum heart rate at induction	112.9 ± 14.69	133.93 ± 13.29	5.81	< 0.001
6.	Emergence delirium score	1.93 ± 0.58	2.83 ± 0.79	5.01	< 0.0001

Incidence of sneezing after IN administration of the study drug was higher in Group K (26.66%) than in Group D (6.66%), but this difference was not significant. The mean score of ED was significantly high (p<0.00001) in Group K (2.83 ± 0.79) than in Group D (1.93 ± 0.58). Incidence of side effects like tachycardia, hypertension, and PONV was higher in Group K, but p value was significant only for PONV (p=0.006). Whereas episode of bradycardia and hypotension was recorded more in Group D, but this difference was not significant (Table 8).

**Table 8:** Incidence of adverse events. PONV, post-operative nausea and vomiting

S. No.	Adverse events	Group K (n=30)	Group D (n=30)	Chi square value with Yates correction	p value
1.	Sneezing and coughing	8 (26.66%)	2 (6.66%)	4.32	0.083
2.	Hypotension	1	4 (13.33%)	0.94	0.33
3.	Hypertension	6 (20%)	3 (10%)	0.52	0.47
4.	Bradycardia	2 (6.66%)	8 (26.66%)	3	0.83
5.	Tachycardia	10 (33.33%)	4 (13.33%)	2.32	0.126
6.	PONV	12 (40%)	2 (6.66%)	7.54	0.006

## Discussion

Separation anxiety is most commonly experienced in children between 1 and 5 years of age and can be reduced effectively by pharmacological interventions [9]. Various routes of premedication have been tried like, oral, intravenous (IV), intramuscular (IM), rectal, and transmucosal. Each drug and route of administration has its own disadvantages. The oral route has low bioavailability, IM and IV routes are invasive and painful, and the rectal route is not convenient. Transmucosal routes like IN and sublingual have shown better compliance [10]. They are more rapid and effective routes of drug administration because of their ability to bypass first-pass metabolism and rich mucosal vascularization [11]. Intranasal administration of drugs can cause nasal irritation, sneezing, and coughing in some children, which can be prevented by using less volume of an undiluted solution of the drug. When a large volume of drug is instilled into the nose, there is a high possibility of entering the drug into the pharynx producing coughing and sneezing. Keeping this in mind, we decided to administer 0.5 mL of study drug solution to each nostril. This might explain the lesser incidence of coughing and sneezing (16.66%), after IN administration of study drugs in our study. Many studies have shown that IN route is an effective way to administer premedication and sedation to children.

Midazolam, ketamine, and dexmedetomidine are the most common intranasally used premedicant in pediatric patients. Ketamine is N-methyl-d-aspartate (NMDA) receptor antagonist and produces dissociative anesthesia. Studies have reported effective and safe use of IN ketamine in doses of 5-6 mg kg^-1^, for premedication in children [12]. But limitations of the use of ketamine are higher incidences of cognitive impairment, PONV, delayed recovery, and increased secretion [13]. Dexmedetomidine is a newer more selective α-2-agonist with a shorter half-life. It has properties to provide sedation, analgesia, and anxiolysis without respiratory depression. Over the past few years, its use has become more prevalent in pediatric clinical practice. Its bioavailability by various routes has made it more popular [14]. Oral dexmedetomidine and ketamine have shown comparable effectiveness as a premedicant with fewer undesirable effects [15]. Due to its colorless and odorless properties, dexmedetomidine is quite suitable for IN administration. IN dexmedetomidine (1 mcg kg-1) is as effective as oral dexmedetomidine (3 mcg kg-1) in achieving a final sedation score with a faster onset of action [16]. IN dexmedetomidine in doses of 1 and 2 mcg kg^-1^ took 20 and 10 min respectively to reach the minimum serum concentration (100 ng mL^-1^) required to achieve desired sedation score. Doubling of IN dose has halved its onset of action. On pharmacokinetic evaluation, it was recorded that peak plasma concentrations of both doses were achieved at 47 min with 84% bioavailability [17]. A study by Sheta et al. also reported that the median time of onset of sedation after IN dexmedetomidine in the dose of 1-2 mcg kg^-1^ was 25-40 min [18]. This was the background behind IN administration of premedication 30 min before transferring the patient into OT in our methodology.

Based on observations of previous literature on the efficacy of IN dexmedetomidine as premedication in children, we have undertaken this study with the hypothesis that IN dexmedetomidine (1 mcg kg-1) would be more effective and with fewer adverse effects than IN ketamine (5 mg kg-1) [19-20]. But we recorded comparable SS15 in both groups and significantly better SS30 in Group K. MAS was also significantly better in Group K. Though, a stable hemodynamic parameter, less incidence of ED and PONV was recorded in Group D.

A randomized comparative study to compare IN dexmedetomidine (2.5 mcg kg^-1^) versus IN ketamine (5 mg kg^-1^) as premedication for the level of sedation in children undergoing radiation therapy demonstrated that dexmedetomidine is superior to ketamine [21]. Ibrahim reported that IN dexmedetomidine is as effective as IN ketamine for procedural sedation to facilitate parental separation and IV cannulation in children undergoing MRI [22]. But their methodology differs from ours as they have used IN dexmedetomidine in a higher dose. This might explain the difference in the outcome.

There are some published trials, where authors have not documented any difference in efficacy of IN ketamine and IN dexmedetomidine premedication, like Gyanesh and colleagues have not found significant differences in children’s response to comparing the efficacy of IN dexmedetomidine (1 mcg kg^-1^) versus IN ketamine (5 mg kg^-1^) premedication for IV placement [23]. In another randomized trial, efficacies of nebulized dexmedetomidine, ketamine, and their combination were compared. No significant difference was found in the ease of parental separation and face mask acceptance in groups where subjects were nebulized with either dexmedetomidine or ketamine alone. The level of sedation was significantly greater in dexmedetomidine-ketamine combination group. Heart rate and mean arterial pressure were found to be significantly lower in dexmedetomidine group [24].

Likewise, we also observed significantly lower minimum and maximum HR in Group D. This can be explained by the fact that dexmedetomidine decreases sympathetic outflow and circulating catecholamine levels [25]. Contrary to dexmedetomidine, ketamine produces its sympathomimetic actions primarily by direct stimulation of central nervous system structures.

On thorough literature search, we found that few authors have proposed the combined use of dexmedetomidine and ketamine for superior sedation, analgesia, premedication, ease of parental separation, and fewer adverse effects due to the opposite effect of both drugs on sympathetic outflow and complimentary pharmacodynamics [26-27].

Unlike conventional sedative drugs which produce their clinical effect by acting on GABA receptors, dexmedetomidine primary site of action in the central nervous system is locus coeruleus, where it induces electroencephalogram activity similar to natural sleep. Hence, dexmedetomidine induces conscious sedation, i.e. patients can be readily aroused by verbal command or light tap. They are also less likely to become disorientated and uncooperative [28]. Dexmedetomidine has a proven role in decreasing the incidence of ED in high-risk pediatric populations [29]. We also observed a significantly lesser mean score of ED in Group D. Incidence of PONV was also less in Group D.

As we have administered dexmedetomidine in a similar dose to all study subjects between 1 and 8 years of age, this could have possibly influenced our results. A randomized comparison of two different doses of IN dexmedetomidine premedication in children was done by Yuen et al. and they concluded that IN dexmedetomidine in doses of 1 and 2 mcg kg^-1^ produced a high rate of satisfactory sedation in children aged 1-4 years, whereas, in children aged 5-8 years, higher doses of IN dexmedetomidine (2 and 3 mcg kg-1) were required [30]. Hence, not implementing age stratification might be a limitation of our study. We chose to instil the study drug by calibrated dropper, as it is simple, convenient, and does not require special training or equipment. But IN administration of the study drugs should have been preferably done by nebulization as converting the drug into an atomized spray increases the ability to cover maximum area and less drug loss to the oropharynx, resulting in better patient acceptability, and clinical efficacy [31].

## Conclusions

Intranasal dexmedetomidine in a dose of 1 mcg kg^-1^ is clinically less effective as premedication in older children as compared to ketamine (5 mg kg^-1^), but associated with fewer incidence of ED and PONV. We recommend the usage of IN dexmedetomidine in a higher dose (1.5-2 mcg kg^-1^), through nebulization/atomizer for the desired level of sedation, mask acceptance with the advantage of lesser incidence of ED and PONV.
